# In hospital falls of a large hospital

**DOI:** 10.1186/s13104-019-4318-9

**Published:** 2019-05-23

**Authors:** Aline Brenner de Souza, Vania Röhsig, Rubia Natasha Maestri, Mohamed Fayeq Parrini Mutlaq, Elisiane Lorenzini, Belisa Marin Alves, Daniela Oliveira, Danusa Cristina Gatto

**Affiliations:** 1 Hospital Moinhos de Vento, Porto Alegre, RS Brazil; 2 Federal University of Santa Catarina, Florianópolis, SC Brazil

**Keywords:** Accidental falls, Aged, Accident prevention, Hospitals, Nursing, Patient safety, Risk management

## Abstract

**Objectives:**

The present database contains information on patient falls in the hospital setting. Data were collected in January 2018 with of describing in-hospital falls reported from 1st January 2012 to 31 December 2017 in a large hospital in the South of Brazil. Learning about the characteristics of these events and establishing a profile may contribute to the design of adequate prevention and improvement strategies that are effective to reduce the risk of falls.

**Data description:**

This data set encompasses 1.071 in-patients falls characterized by the follow variables: year, date, patient birth, weekday, shift, department/location of the incident, location, severity, presence of companion, age, sex, risk level, medication associated with fall risk, implemented fall prevention protocol, type of injury, reason, restraint prescription, physical therapy prescription.

## Objective

Falls, defined by the World Health Organization as events “which result in a person coming to rest inadvertently on the ground or floor or other lower level,” are the second leading cause of accidental or unintentional deaths in the world. In-hospital fall rates have been reported to range from 1.3 to 16.9 per 1000 patient days, with a negative impact on health systems resulting from the associated increase in mortality rates, hospital length of stay, and admission costs, as well as decreased quality of life [1–3].

In-hospital falls are avoidable accidents, but continue to be a high prevalent patient safety issue with a negative impact on health systems. The reporting of falls in hospital settings is recognized as a prevention strategy worldwide. Incidents are reported via institution-based or nationwide patient safety incident reporting systems in various countries. The aim of these systems should not be on remediating the incident itself, but rather on the ability of designing corrective (often systemic) actions that are truly appropriate to prevent future events [4–7]. However, because falls are probably underreported, most estimates are likely to be underestimated. Indeed, little is known about the associated factors of in-hospital falls. It should be noted that the hospital where we gathered this data set consistently invests in establishing a culture of safety, and thus underreporting of cases is unlikely. Then, the high number of fall reports in this hospital may reflect the different definitions of fall available in the literature, which include both the need to reach the ground or simply the need for support, even if the individual does not come to rest on the ground [8]. In addition, the standards for accreditation may also explain, at least in part, the high number of reports.

The present database contains 1071 reports of patient falls in the hospital setting. Data were collected in January 2018 with the aim of describing in-hospital falls reported from 1st January 2012 to 31 December 2017 in a large hospital in the South of Brazil. Learning about the characteristics of these events and establishing a profile may contribute to the design of adequate prevention and improvement strategies that are effective to reduce the risk of falls. The analysis of all reports is essential to identify risk groups and to implement measures that aim to avoid the falls associated with serious injury. A contribution of the present dataset is that their analyses can generate falls prevention strategies to be implemented primarily with individuals identified as being at high risk of falls.

Also, upon this data set, a pilot study testing all desired interventions can be performed, which must be adjusted to the context to which they will be applied using knowledge translation strategies. Thus, stakeholders must be identified and involved in the design of programs that include multifaceted, multidisciplinary interventions to promote the safety of patients regarding falls prevention.

## Data description

This data set (Table 1) was obtained from the Office for Risk Management at Hospital Moinhos de Vento (HMV) a 497-bed institution located in the city of Porto Alegre, South of Brazil. HMV is one among only six hospitals in Brazil to be rated as a Center of Excellence by the Ministry of Health. It was also the first hospital in the South region to be accredited by the Joint Commission International, in 2002. Organizational planning and a strong safety culture are important aspects of the institution’s strategic plan. In this hospital, all actions are guided by the search for excellence, and the institution has invested in continuous improvement to enhance the quality and safety of the care provided.

**Table 1:** Overview of data files/data sets

Label	Name of data file/data set	File types (file extension)	Data repository and identifier (DOI or accession number)
*Data file 1*	*In*-*hospital falls DATA from 2012*–*2017*	*In*-*hospital falls: data set (2012–2017).xlsx*	10.6084/m9.figshare.7387679.v1

The incident reporting system was implemented at the hospital in 1st January 2012. All falls are reported in the hospital’s incident reporting system as soon as they occur by frontline staff. The HMV Office for Risk Management regularly monitors all falls reported in the hospital’s reporting system by reviewing the electronic report form. Data collection was performed in January 2018. An Excel spreadsheet was generated. No exclusion criteria were applied, and therefore, all falls reported from 1st January 2012 to 31 December 2017 were included.

This data set encompasses 1.071 in-patients falls characterized by the follow variables: year, date, patient birth, weekday, shift, department/location of the incident, location, severity, presence of companion, age, sex, risk level, medication associated with fall risk, implemented fall prevention protocol, type of injury, reason, restraint prescription, physical therapy prescription.

This data can be analyzed by using descriptive statistics and presented as measures of central tendency (mean and median) and variability (standard deviation and interquartile range), as well as absolute and relative distributions (n − %) can also reported. The symmetry of continuous distribution can be assessed by the Kolmogorov-Smirnov test.

To compare intra-variable category proportions (univariate analysis) the Pearson’s Chi square test for homogeneity can be used, complemented by adjusted residual analysis, in which estimates equal to or above |1.96| indicate a significant difference between the categories being compared. For comparison of two independent categorical variables, Pearson’s Chi square test can be used. Also, multivariate logistic regression analysis, odds ratio and relative risk analyses can be performed.

## Limitations

The limitation of this data set is the lack of description about which medications associated with the risk of falls [9] were being used by patients who have falls. The description of the medication is not usually informed in the incident report. However, the lack of this analysis does not compromise the data set, since this database included an expressive number of incidents and other variables of interest can be extensively explored.

## Data Availability

The datasets generated during and/or analysed during the current study are available in the figshare repository: 10.6084/m9.figshare.7387679.

## Abbreviation

HMV: Hospital Moinhos de Vento
